# Benefits and costs of a hypercapsule and the mechanism of its loss in a clinical isolate of *Acinetobacter baumannii*

**DOI:** 10.1128/mbio.02366-25

**Published:** 2025-12-29

**Authors:** Chaogetu Saren, Ken-Ichi Oinuma, Taishi Tsubouchi, Arata Sakiyama, Masato Suzuki, Mamiko Niki, Yukihiro Kaneko

**Affiliations:** 1 Department of Bacteriology, Osaka City University Graduate School of Medicine 198295 , Osaka, Japan; 2 Department of Bacteriology, Osaka Metropolitan University Graduate School of Medicine 198295 , Osaka, Japan; 3 Research Center for Infectious Disease Sciences, Osaka Metropolitan University Graduate School of Medicine 12935, Osaka, Japan; 4 Laboratory of Phage Biologics, Graduate School of Medicine, Gifu University 12785, Gifu, Japan; 5 Antimicrobial Resistance Research Center, National Institute of Infectious Diseases 13511, Tokyo, Japan; 6 Osaka International Research Center for Infectious Diseases, Osaka Metropolitan University 12936, , Osaka, Japan; Universiteit Gent, Gent, Belgium

**Keywords:** *Acinetobacter baumannii*, hypercapsule, capsule switching, phenotypic heterogeneity, insertion sequence

## Abstract

*Acinetobacter baumannii* is an opportunistic pathogen in which capsule production is closely linked to immune evasion and environmental persistence. Recent studies have described two seemingly contradictory phenomena—increasing prevalence of capsule-overproducing clinical isolates and frequent isolation of capsule-deficient variants. The biological significance of these phenomena remains unclear. In this study, we analyzed a clinical isolate, OCU_Ac16b, which spontaneously gives rise to two phenotypically distinct variants: the L type forming large colonies with a prominent hypercapsule (>1 µm thick), and the S type forming small colonies with a substantially reduced or absent capsule. When L-type cells were cultured in test tubes under low-shaking conditions, S-type variants reproducibly emerged, constituting approximately 40%–80% of the population within 24 h. Whole-genome sequencing revealed that this conversion is driven by distinct mutations in the capsular polysaccharide synthesis cluster, including insertion sequence insertions and a single-nucleotide deletion. Dilution experiments demonstrated that L-to-S conversion mutations arise *de novo* during liquid culture at sufficiently high rates to account for the rapid and reproducible emergence of S-type variants. Phenotypic characterization demonstrated a biological trade-off, with L-type cells exhibiting enhanced resistance to serum killing, desiccation, and certain β-lactam antibiotics, whereas S-type cells showed superior surface attachment, increased biofilm formation, and a growth advantage under oxygen-limited conditions. Our findings uncover a highly reproducible, mutation-driven capsule switching mechanism that enables rapid phenotypic adaptation to changing environments. This phenotypic heterogeneity has significant implications for pathogenesis, persistence, diagnostic evaluation, and clinical management.

**IMPORTANCE:**

*Acinetobacter baumannii* is a clinically important opportunistic pathogen that exhibits striking phenotypic diversity. In particular, some clinical isolates produce unusually thick capsules, which are thought to contribute to immune evasion and persistence, while others lack the capsule altogether. However, the biological significance of these contrasting phenotypes has remained unclear. We analyzed a clinical isolate that spontaneously gives rise to capsule-deficient variants from a hypercapsulated form. We found that the conversion is driven by spontaneous mutations in capsule biosynthesis genes, including *de novo* mutations arising during liquid culture, while the expansion of capsule-deficient cells is promoted under oxygen-limited conditions. The two variants differed in serum resistance, desiccation tolerance, growth characteristics, and antibiotic responses, revealing a trade-off between protective barriers and environmental adaptability. These findings provide new insights into how *A. baumannii* may balance survival strategies through genetic and phenotypic heterogeneity, with potential implications for diagnosis, treatment, and bacterial persistence in clinical settings.

## INTRODUCTION

*Acinetobacter baumannii* is an opportunistic gram-negative pathogen that has emerged as a major cause of hospital-acquired infections worldwide. *A. baumannii* infections commonly occur in immunocompromised patients and are associated with ventilator-associated pneumonia, bloodstream infections, wound infections, and urinary tract infections (1–3). A defining characteristic of *A. baumannii* is its ability to rapidly acquire antimicrobial resistance through a wide array of mechanisms, including horizontal gene transfer via plasmids and natural transformation (1, 2, 4, 5). The genome of *A. baumannii* harbors numerous insertion sequences (ISs), which are minimal transposable elements encoding transposase. ISs often enhance the expression of chromosomal resistance genes, such as *bla*_OXA-51_, by inserting strong promoter sequences (6, 7). In addition, ISs can disrupt various genes critical for antimicrobial susceptibility, such as those encoding outer membrane porins (7, 8).

Beyond resistance, *A. baumannii* exhibits a remarkable adaptability to diverse environments, in part facilitated by biofilm formation and capsule production (9). Capsules have been shown to contribute to immune evasion, disinfectant resistance, and desiccation tolerance, making them a significant determinant of bacterial pathogenicity (10). A recent study reported that an increasing number of modern clinical isolates of *A. baumannii* display thicker capsules and higher levels of capsulation compared to historically established strains (11). Other studies have described the rising prevalence of mucoid *A. baumannii* isolates, which exhibit markedly thickened capsules and are often associated with increased virulence (12, 13). In addition, mucoid variants have been reported in the context of long-term or persistent infections, particularly among patients undergoing prolonged hospitalization with invasive medical procedures (12). However, the underlying mechanisms and biological consequences of this phenotypic shift remain largely unexplored.

We recently reported on the clinical isolation and genomic characterization of *A. baumannii* OCU_Ac16a, harboring *bla*_NDM-1_, *bla*_TMB-1_, and *bla*_OXA-58_, and its clone, OCU_Ac16b, which lacks *bla*_NDM-1_ but retains *bla*_TMB-1_ and *bla*_OXA-58_ (14). OCU_Ac16a and OCU_Ac16b were isolated from sputum cultures of a postoperative patient with esophageal cancer. While our initial focus was on their antimicrobial resistance, subsequent observations made during the course of this study revealed that OCU_Ac16b exhibits notable variations in colony size and capsular morphology. More specifically, a subpopulation of OCU_Ac16b exhibited a remarkably thick capsule (hypercapsule), forming relatively large colonies on agar plates (designated the L type), while other variants with either no capsule or a much thinner one formed smaller colonies (designated the S type). Moreover, we found that S-type variants emerged within the L-type population during liquid culture, replacing 40%–80% of the population within 24 h. This phenomenon was consistently observed in repeated independent experiments, with near-complete reproducibility.

In this study, we aimed to elucidate the clinical relevance and mechanistic basis of this L-to-S phenotypic conversion in OCU_Ac16b. Through genome sequencing of representative L- and S-type variants, we found that the conversion is driven by distinct mutations, such as multiple IS insertions and at least one single-nucleotide deletion, within the capsular polysaccharide synthesis (*cps*) cluster. We further found that *cps*-disrupting mutations arise *de novo* during liquid culture at sufficiently high frequencies to account for the rapid and reproducible emergence of S-type variants. These findings highlight a previously unrecognized phenotypic switch in *A. baumannii*, driven by frequent mutations arising at multiple sites in the *cps* cluster that lead to capsule loss. Our findings also shed light on the functional trade-offs associated with hypercapsule production: while L-type cells exhibit enhanced resistance to serum killing, desiccation, and certain β-lactam antibiotics, S-type variants demonstrate superior surface attachment, biofilm formation capacity, and a fitness advantage under oxygen-limited conditions. By demonstrating that these genetic changes confer distinct advantages and disadvantages under different environmental conditions, this study illustrates the dual nature of hypercapsule formation as both a protective and burdensome trait. Our study underscores the importance of considering dynamic and heterogeneous bacterial subpopulations when evaluating virulence, resistance, and treatment outcomes.

## RESULTS

### Discovery and characterization of L-type and S-type variants of OCU_Ac16b

**Fig 1:**
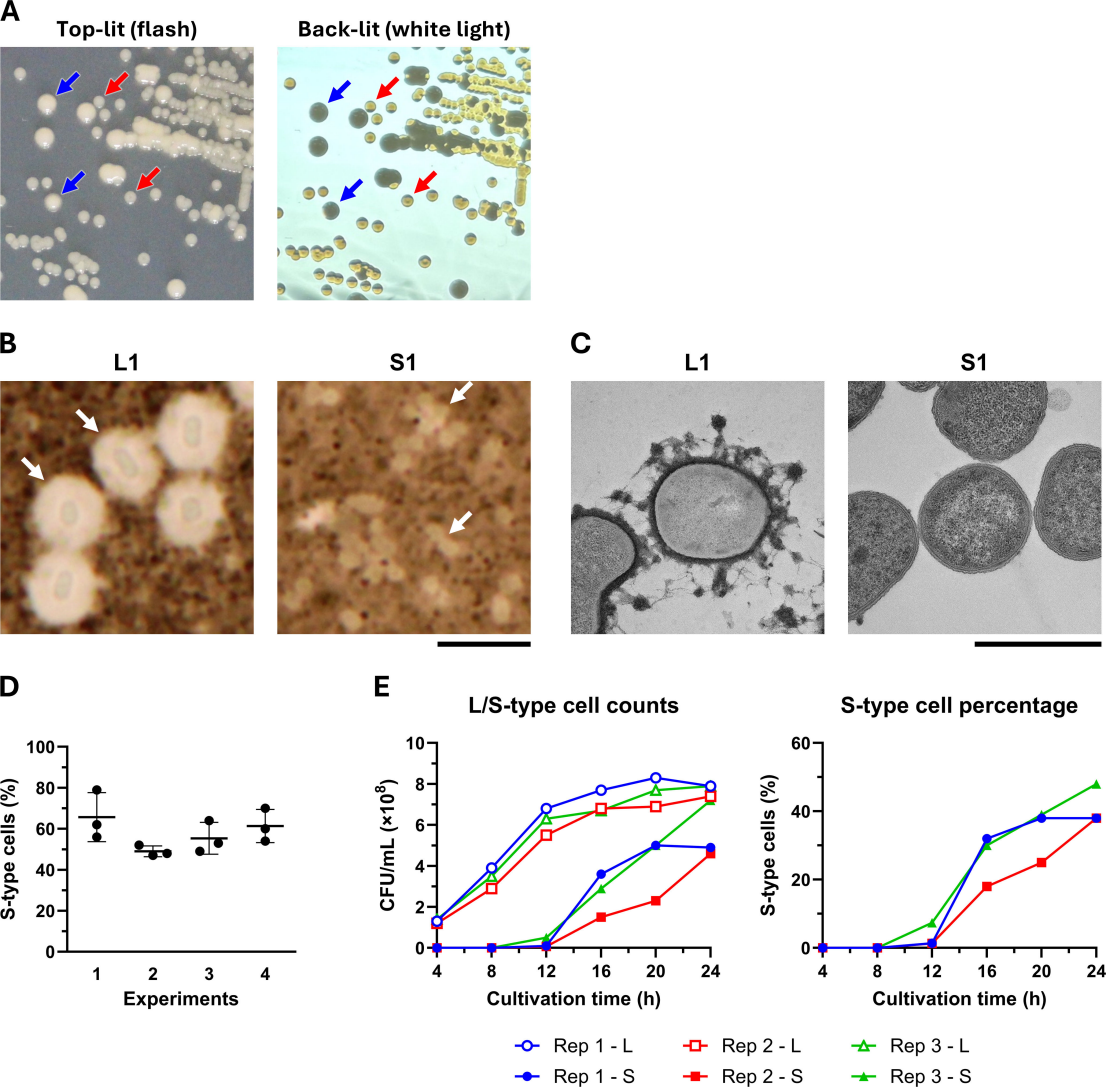
Discovery and characterization of L-type and S-type variants of *Acinetobacter baumannii* OCU_Ac16b. (**A**) OCU_Ac16b forming both L-type and S-type colonies was grown on CAMH agar plates overnight at 37°C. The left panel shows colonies photographed from above with flash illumination. The right panel shows colonies photographed with back illumination; the plate was held approximately 50 cm in front of a white light source. Representative L-type (blue arrows) and S-type (red arrows) colonies are indicated. (**B**) Negative staining with India ink of L1 (L type) and S1 (S type) cells. Cells were observed under a light microscope. L1 cells exhibited a prominent halo approximately 1.0–1.5 μm thick, indicating the presence of a hypercapsule, whereas S1 cells showed no visible capsule. Arrows indicate representative cells. Scale bar, 5 μm. (**C**) TEM of L1 (L type) and S1 (S type) cells. Cells were stained with ruthenium red to visualize capsular polysaccharides. L1 cells displayed an electron-dense capsular layer with irregular, fibrous extensions surrounding the cell, whereas S1 cells showed no detectable capsule structure. Scale bar, 1 μm. (**D**) Conversion of L-type to S-type variants during liquid culture. Single colonies of the L-type strain (L1) were inoculated into 3 mL of CAMH broth in 14-mL test tubes and incubated at 37°C with shaking at 120 rpm for 24 h. Cultures were then plated onto CAMH agar plates to evaluate the colony morphologies. Four independent experiments were performed, each using three separate colonies. (**E**) Time course of S-type cell emergence from L-type cultures. Three independent L1 cultures (Rep 1 to 3) were grown in CAMH broth (3 mL, 37°C, shaking at 120 rpm), and samples were taken at indicated time points to determine colony-forming units (CFUs) of L-type and S-type cells. The left graph shows the CFUs for each type, and the right graph indicates the percentage of S-type cells in the population.

To investigate the phenotypic heterogeneity of OCU_Ac16b, we selected representative L-type and S-type variants (designated L1 and S1, respectively) and compared their colony morphology and capsule production. On cation-adjusted Mueller Hinton (CAMH) agar plates, L-type colonies appeared approximately 1.5 times larger and more opaque than S-type colonies (Fig. 1A). Light microscopy with India ink-negative staining revealed a prominent hypercapsule surrounding L1 cells, with capsular layer thickness ranging from approximately 1.0 to 1.5 μm, whereas no visible capsule was observed in S1 cells (Fig. 1B). Transmission electron microscopy (TEM) with ruthenium red labeling further confirmed these observations, showing an electron-dense capsular layer with tangled, fibrous extensions in L1 cells, while S1 cells lacked any detectable capsule-like structure (Fig. 1C).

During liquid culture, S-type colonies reproducibly emerged from L-type populations. When single L-type colonies were inoculated into 3 mL of CAMH broth in 14-mL tubes and incubated at 37°C with shaking at 120–130 rpm for 24 h, S-type cells comprised approximately half of the population. This conversion was unidirectional as S-type cultures did not yield L-type variants. The phenomenon was unlikely to be due to contamination as S-type colonies were absent when L1 stocks were directly plated or when L-type colonies were transferred to agar without liquid culture. To assess reproducibility, we conducted four independent L-type culture experiments, each with triplicate cultures under standardized conditions (CAMH broth, 37°C, 120 rpm). S-type cells consistently emerged, comprising 47%–79% of the population after 24 h (Fig. 1D). Time-course analysis of three independent L1 cultures showed that S-type cells were not detected at 8 h but became detectable in all cultures by 12 h, reaching 38%–48% by 24 h (Fig. 1E). These results demonstrate reproducible L-to-S phenotypic conversion during liquid culture.

### Identification of genetic determinants of L-to-S phenotypic conversion

**Fig 2:**
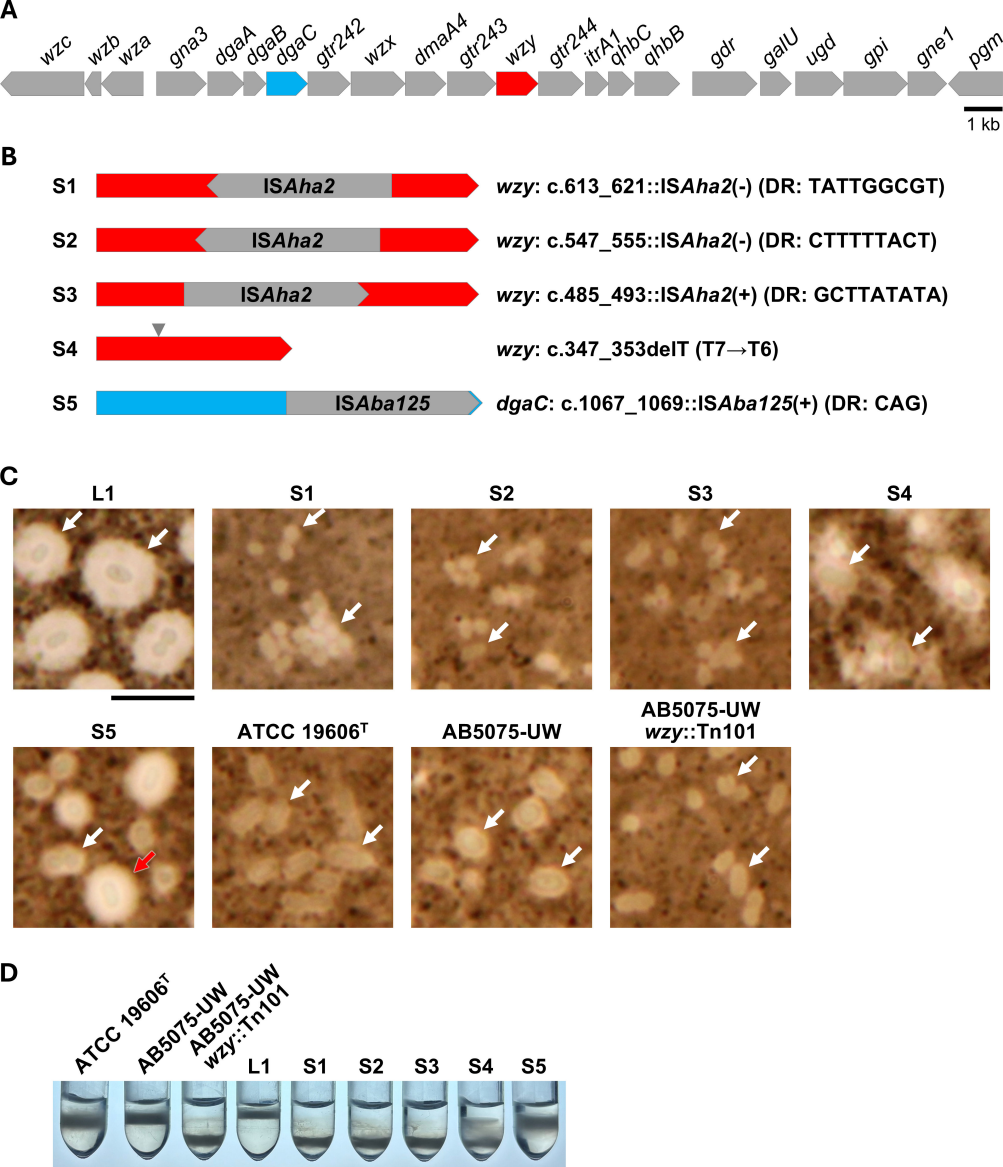
Identification of mutations responsible for the phenotypic conversion from L type to S type in *Acinetobacter baumannii* OCU_Ac16b. (**A**) Schematic representation of the entire capsule polysaccharide synthesis gene cluster in L1. *wzy* and *dgaC* are highlighted in red and light blue, respectively. (**B**) Schematic representation of genetic alterations identified in S-type variants S1–S5. S1–S4 harbor mutations in *wzy*, while S5 contains an insertion in *dgaC*. “c.” indicates the position within the coding sequence of the gene. (+) and (−) indicate that an IS is oriented in the same or opposite direction, respectively, relative to the target gene. DR, direct repeat sequence observed at both ends of the insertion elements. (**C**) Capsule phenotypes of L1 and five S-type variants (S1–S5), as well as ATCC 19606^T^, AB5075-UW, and AB5075-UW *wzy*::Tn101, visualized by negative staining with India ink and light microscopy. S1–S3 lacked visible capsule structures and frequently formed small aggregates. S5 showed both thinly and thickly encapsulated cells with clearly defined capsule boundaries, whereas S4 displayed irregular, variably sized halos lacking sharp boundaries, consistent with a diffuse or unstable capsule-associated matrix. Representative cells are indicated with white arrows. In S5, a red arrow highlights a thickly encapsulated cell within the heterogeneous population. Scale bar, 5 μm. (**D**) Density gradient centrifugation of *A. baumannii* variants. Cells from ATCC 19606^T^, AB5075-UW, AB5075-UW *wzy*::Tn101, and OCU_Ac16b-derived variants (L1 and S1–S5) were layered onto 40% Percoll and centrifuged. The strains formed distinct bands at different positions, reflecting differences in buoyant density associated with capsule abundance.

Genome comparison between L1 and S1 strains revealed an 875-bp inversion at the *brnT*/*brnA* locus (positions 7,007–7,881 of plasmid pOCU_Ac16a_3), which is presumed unrelated to capsule switching. More importantly, S1 harbored a 1,040-bp IS (IS*Aha2*) within the *wzy* gene encoding polysaccharide polymerase in the *cps* cluster (Fig. 2A and B). The insertion at positions 613–622 of *wzy* created a 9-bp direct repeat (TATTGGCGT) flanking the IS element, with the transposase oriented opposite to *wzy*. This *wzy* disruption likely accounts for the capsule loss in S1.

To assess the reproducibility of this mutation event, we isolated 16 S-type colonies newly emerged from an L-type culture and screened them by PCR for IS insertions in *wzy*, using a primer pair listed in Table 1. Four of the 16 strains showed PCR product sizes indicative of IS insertion. Subsequent sequencing of the putative IS-inserted PCR products revealed two additional IS*Aha2* insertions at distinct sites within *wzy*, found in strains S2 and S3 (Fig. 2B). We selected four representative strains for further analysis: S2 and S3, which carried IS*Aha2* insertions in *wzy*, and S4 and S5, which lacked such insertions. Genome sequencing revealed no additional mutations in S2 and S3 beyond the IS*Aha2* insertions in *wzy*. S4 had a single thymine base deletion in *wzy*, and S5 harbored an IS*Aba125* insertion in *dgaC*, a putative aminotransferase gene located within the *cps* cluster (Fig. 2B). These findings suggest that spontaneous mutations in key *cps* genes are the direct cause of L-to-S phenotypic conversion in most cases, if not all.

**TABLE 1:** Primers used in this study

Primer name	Sequence (5'→3')	Application/notes
wzy_check_F	GCTGGTAGAGATGAGCAGG	PCR to detect insertions in *wzy* based on the amplicon size
wzy_check_R	TCCGAGGTAAAGGCATAAAC	Used both in insertion detection PCR and as a nested PCR reverse primer
Nested_wzy::ISAha2(+)_F1	GCACTGCAGACAAGTCATTC	First round PCR to detect IS*Aha2* in forward orientation
Nested_wzy::ISAha2(+)_F2	TCCTCATACCCAAGTTGTCAC	Second round (nested) PCR for forward orientation
Nested_wzy::ISAha2(−)_F1	AGCGTACTGTAATCTGGTGC	First round PCR to detect IS*Aha2* in reverse orientation
Nested_wzy::ISAha2(−)_F2	AGCGCGATTATAGGAAGACC	Second round (nested) PCR for reverse orientation
Nested_wzy::ISAha2(±)_R1	GGTAGGTGGCCTTAATATCTGC	First round PCR reverse primer (common to both orientations)
Nested_wzy::ISAha2(±)_R2	TCCGAGGTAAAGGCATAAAC	Second round PCR reverse primer (same as wzy_check_R)

Microscopy confirmed that S2 and S3, like S1, lacked visible capsule structures and tended to form small aggregates, whereas S4 and S5 exhibited extracellular material surrounding the cells and were more often observed as dispersed single cells (Fig. 2C). In S5, this material appeared as variably sized, clearly demarcated capsule layers, consistent with heterogeneous capsule production. In contrast, the material surrounding S4 cells was markedly more diffuse and irregular, frequently lacking a clearly defined outer boundary, suggesting altered or unstable capsule-associated matrix rather than a well-formed capsule. Density gradient centrifugation corroborated these observations (Fig. 2D). L1 localized near the top of the gradient, reflecting low buoyant density likely due to its thick capsule, while S1–S3 sedimented to lower positions consistent with capsule loss. S4 formed a major band at the same position as S1–S3 with a faint upward smear, whereas S5 occupied an intermediate position with a broadened band. The pattern observed for S5 is consistent with heterogeneous capsule production. In contrast, the banding pattern of S4 likely reflects the presence of irregular or loosely associated extracellular material rather than a well-defined capsule layer.

### The clinical relevance of the L-to-S phenotypic conversion

To assess the clinical relevance of capsule switching, we compared the antimicrobial susceptibility, serum resistance, desiccation tolerance, and biofilm formation of L-type and S-type variants. Antimicrobial susceptibility testing revealed that L1 cells showed higher resistance to certain β-lactam antibiotics, with minimum inhibitory concentrations (MICs) twice as high for imipenem and more than four times higher for cefepime compared to S1 cells (Table 2). This suggests that the hypercapsule provides protective effects against specific β-lactams.

**TABLE 2:** MICs of 12 antibiotics against *A. baumannii* strains^*a*^

Strain	AMP	PIP	CAZ	FEP	IPM	MEM	KAN	AMK	LVX	MIN	TGC	CST
ATCC 19606^T^	>256	16	4	8	0.25	0.25	8	16	0.25	0.064	1	0.094
L1	>256	>256	>256	>256	16	>32	4	4	0.5	0.064	0.25	0.125
S1	>256	>256	>256	64	8	>32	4	4	0.5	0.047	0.25	0.094

^*a*^ MIC values are given in µg/mL. AMP, ampicillin; PIP, piperacillin; CAZ, ceftazidime; FEP, cefepime; IPM, imipenem; MEM, meropenem; KAN, kanamycin; AMK, amikacin; LVX, levofloxacin; MIN, minocycline; TGC, tigecycline; CST, colistin. All MICs were determined using Etest (bioMérieux).

**Fig 3:**
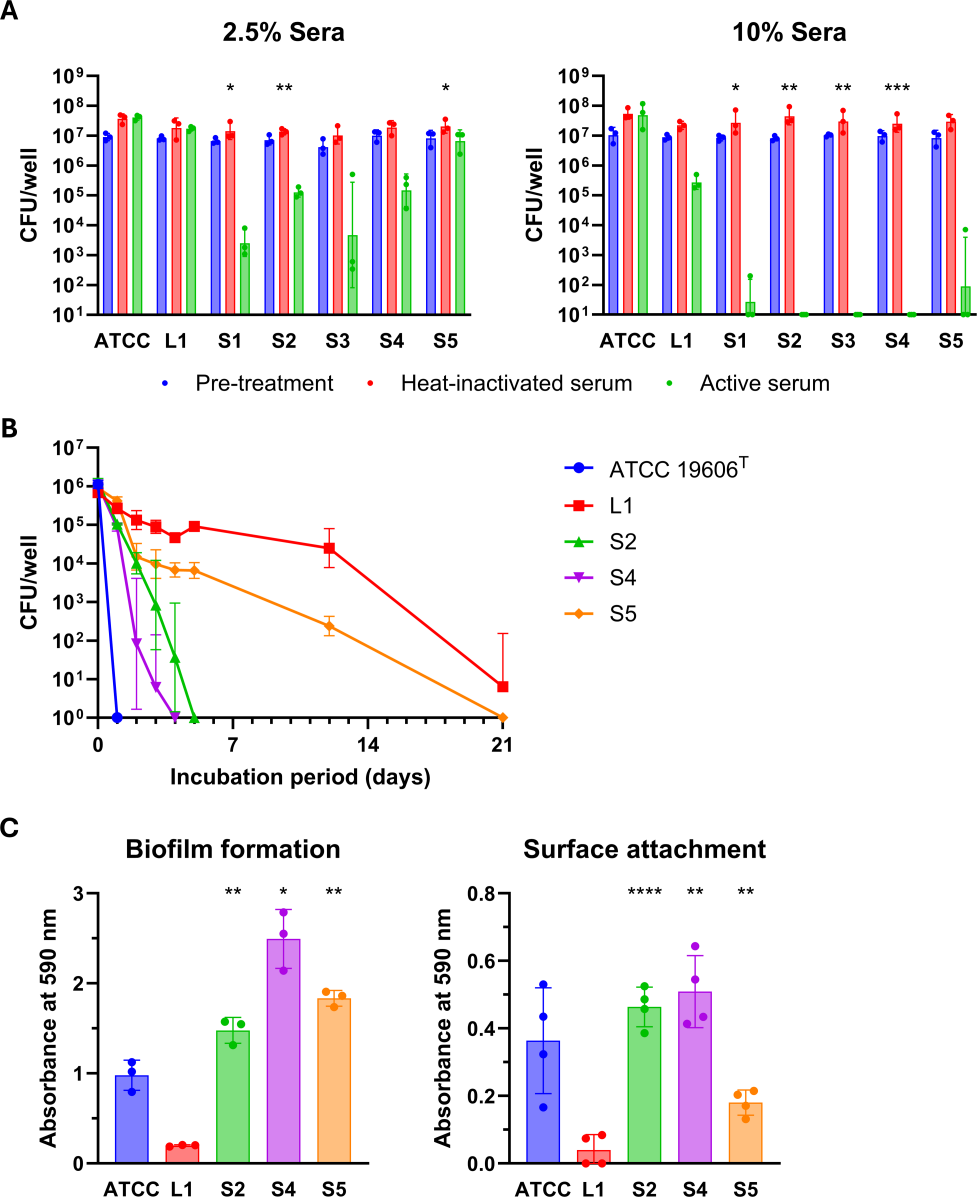
Phenotypic characterization of L-type and S-type variants reveals functional trade-offs associated with capsule production. (**A**) Serum resistance assay. Strains ATCC 19606^T^ (ATCC), L1, and S1–S5 were tested for survival in pooled human serum at final concentrations of 2.5% (left panel) and 10% (right panel). Cell suspensions were incubated in serum at 37°C with shaking at 100 rpm for 2 h. Blue bars represent CFUs prior to serum exposure, red bars represent CFUs after incubation in heat-inactivated serum, and green bars represent CFUs after incubation in active serum. Data represent geometric means ± geometric SD from three biological replicates (independent cultures initiated from single colonies), with individual data points shown. Values below the detection limit (10 CFUs/well) were set to 10 for visualization on a log scale and statistical analysis. Statistical significance among L1 and S1–S5 was assessed by calculating CFU survival ratios relative to pretreatment values, log10-transforming the data, and performing Welch’s one-way analysis of variance (ANOVA) followed by Dunnett T3 multiple comparisons test with L1 as the control. Asterisks indicate significant differences compared with L1 (*, *P* < 0.05; **, *P* < 0.01; ***, *P* < 0.001). (**B**) Desiccation tolerance assay. Strains ATCC 19606^T^, L1, S2, S4, and S5 were tested for survival under desiccating conditions at 36°C and 13.1% (SD 2.8) relative humidity. Cell suspensions were spotted in 96-well plates and incubated under desiccating conditions. At designated time points (1, 2, 3, 4, 5, 12, or 21 days), cells from separate wells were rehydrated and plated to determine viable CFU. Data represent geometric means ± geometric SD from three independently prepared cultures, each derived from a different single colony. Values below the detection limit (1 CFU/mL) were plotted as 1 for visualization on a log scale. Lower error bars are not shown for L1 (day 21) and S4 (day 3) because they fall below the lower limit of the log-scaled Y axis (1 CFU/mL). (**C**) Biofilm formation and surface attachment assays. Strains ATCC 19606^T^, L1, S2, S4, and S5 were evaluated. Left panel: Biofilm formation was assessed after 48 h of static cultivation in Brain-Heart Infusion (BHI) broth at 37°C in 24-well polystyrene plates. Right panel: Surface attachment was evaluated after 2 h of cultivation in CAMH broth at 37°C with shaking at 100 rpm. Both assays used crystal violet staining, and the absorbance was measured at 590 nm. Data represent means ± SD from three or four biological replicates, with individual data points shown. Statistical significance among L1, S2, S4, and S5 was assessed by Welch’s one-way ANOVA followed by Dunnett T3 multiple comparisons test with L1 as the control. Asterisks indicate significant differences compared with L1 (*, *P* < 0.05; **, *P* < 0.01; ****, *P* < 0.0001).

To evaluate the impact of L-to-S phenotypic conversion on serum resistance, we tested the survival of L1, S1–S5, and ATCC 19606^T^ strains in pooled human serum at final concentrations of 2.5% and 10% (Fig. 3A). L1 exhibited high resistance, whereas all S-type variants showed substantially reduced survival, indicating that capsule loss or reduction generally compromises serum resistance. Among the S-type strains, S5 displayed relatively higher survival, consistent with its retention of clearly defined, variably thick capsule layers, while S4, which possesses only diffuse and structurally unstable capsule-associated material, showed markedly lower resistance. These findings suggest that serum resistance depends on both capsule thickness and structural integrity and that reduction in either or both properties compromises protection. Notably, ATCC 19606^T^ also exhibited high serum resistance despite possessing only a thin capsule. This implies the involvement of capsule-independent mechanisms, such as recruitment of complement regulatory factors by outer membrane proteins (15).

We next evaluated the desiccation tolerance of L1 and selected S-type variants (S2, S4, and S5), using ATCC 19606^T^ as a reference (Fig. 3B). L1 cells exhibited the highest tolerance, with viable cells detectable even after prolonged desiccation. In contrast, S-type variants showed markedly reduced survival, and ATCC 19606^T^ was the most sensitive. S5, which retained a capsule of heterogeneous thickness, showed intermediate tolerance. Interestingly, S4, despite retaining the capsule-associated matrix, showed desiccation sensitivity comparable to or even greater than that of the non-encapsulated strain S2. This suggests that the diffuse and irregular material surrounding S4 cells does not confer the protective properties typically associated with an intact capsule. These findings underscore the importance of capsule structural integrity, not merely its presence or thickness, for effective protection against desiccation.

We evaluated biofilm formation in L1 and three selected S-type variants (S2, S4, and S5), with ATCC 19606^T^ included for comparison. After 48 h of static cultivation in BHI broth, all S-type strains produced more biofilms than L1 (7.5- to 12.7-fold), as did ATCC 19606^T^ (5-fold) (Fig. 3C, left). We also found that S2, S4, S5, and ATCC 19606^T^ exhibited a noticeably greater adherence than L1 (Fig. 3C, right), suggesting that enhanced attachment contributes to increased biofilm production. These findings underscore that L-to-S conversion may promote environmental persistence and influence infection outcomes by enhancing biofilm formation and surface colonization.

### S type has a fitness advantage under oxygen-limited conditions

**Fig 4:**
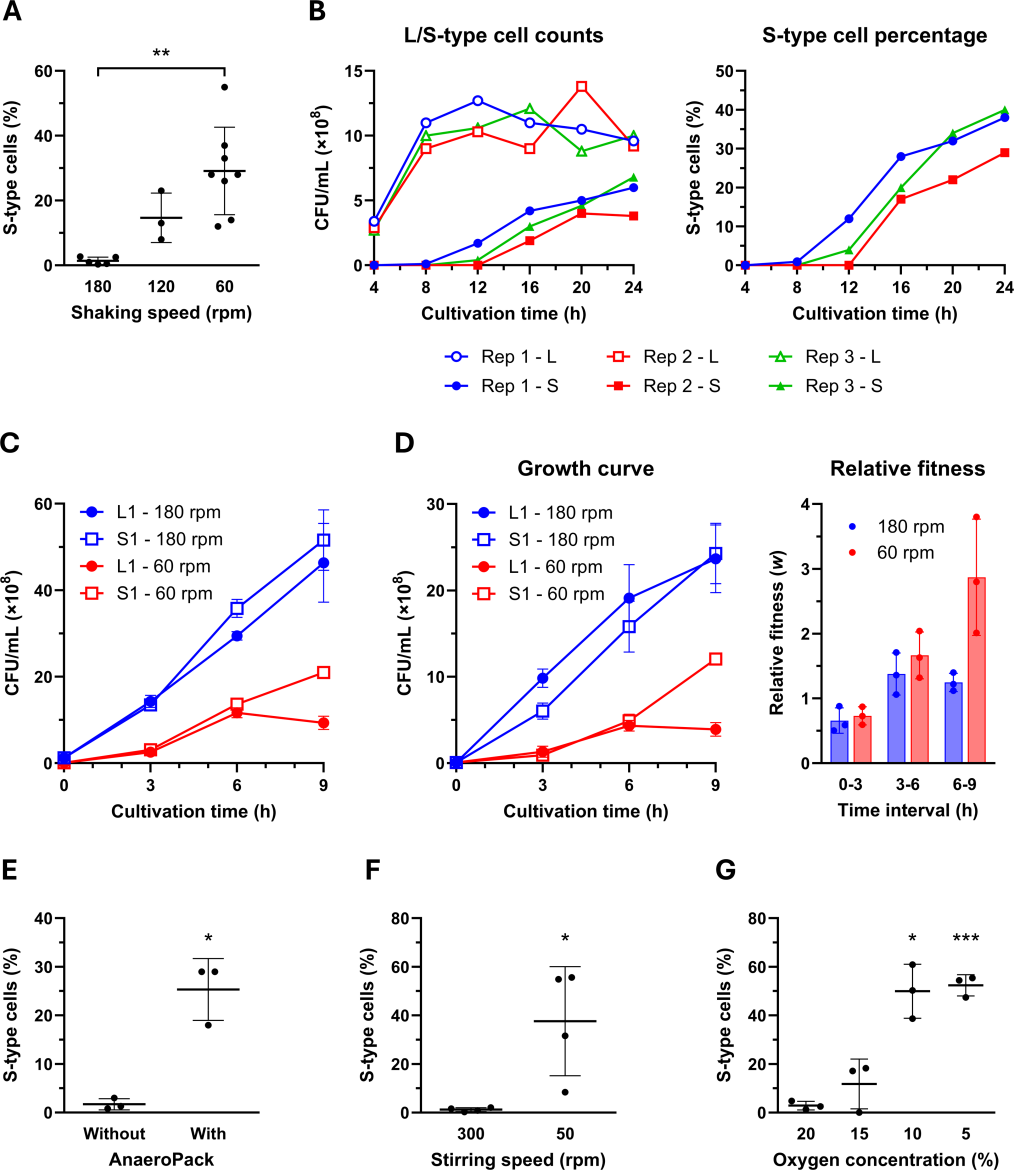
S-type cells have a fitness advantage under oxygen-limited conditions. (**A**) Effect of shaking speed on L-to-S conversion in Erlenmeyer flasks. Single colonies of the L1 strain were inoculated into 10 mL of CAMH broth in 50-mL Erlenmeyer flasks and incubated at 37°C for 24 h at the indicated shaking speeds. Data represent means ± SD from five replicates at 180 rpm, three at 120 rpm, or eight at 60 rpm, with individual data points shown. Statistical significance was assessed by Welch’s one-way ANOVA followed by Dunnett T3 multiple comparisons test. A significant difference was observed only between cultures shaken at 180 rpm and those at 60 rpm (**, *P* < 0.01). (**B**) Time course of S-type cell emergence at low shaking speed. Three independent L1 cultures (Rep 1 to 3) were grown in CAMH broth (10 mL in 50-mL flasks) at 37°C with shaking at 60 rpm. Samples were taken at indicated time points to determine the CFUs of L-type and S-type cells. The left graph shows the CFUs for each type, and the right graph indicates the percentage of S-type cells in the population. (**C**) Growth curves of L1 and S1 strains in monoculture under different shaking conditions. Cultures were grown in CAMH broth (10 mL in 50-mL flasks) at 37°C with shaking at either 180 rpm or 60 rpm. Data represent means ± SD from three biological replicates. (**D**) Competition assay between L1 and S1 strains under different shaking conditions. L1 and S1 pWH1266 cells were mixed at a 1:1 ratio and cultivated in CAMH broth (10 mL in 50-mL flasks) at 37°C for 9 h with shaking at either 180 rpm or 60 rpm. The left panel shows growth curves for both strains under the two conditions. The right panel shows the relative fitness (*w*) of S1 cells with respect to L1 cells, calculated for each time interval (0–3 h, 3–6 h, and 6–9 h). Data represent means ± SD from three biological replicates. Statistical significance for the relative fitness data (right panel) was assessed by two-way repeated measures ANOVA with Geisser–Greenhouse correction. The main effects of shaking speed and time were significant (*P* = 0.022 and 0.013, respectively), while their interaction was not (*P* = 0.071). (**E**) Effect of microaerophilic conditions on L-to-S conversion. Single colonies of the L1 strain were inoculated into 10 mL of CAMH broth in 50-mL flasks and incubated at 37°C with shaking at 180 rpm for 24 h under two conditions: “Without” represents cultivation under ambient air, and “With” represents cultivation in sealed pouches containing AnaeroPack MicroAero to generate microaerophilic conditions. Data represent means ± SD from three biological replicates, with individual data points shown. Statistical significance between the two groups (without and with AnaeroPack) was assessed by Welch’s *t*-test (*, *P* < 0.05). (**F**) Effect of stirring speed on L-to-S conversion. Single colonies of the L1 strain were inoculated into 10 mL of CAMH broth in 50-mL Erlenmeyer flasks containing magnetic stir bars and incubated at 37°C for 24 h with stirring at either 50 rpm or 300 rpm. The proportion of S-type cells was determined by plating and colony counting. Data represent means ± SD from four biological replicates, with individual data points shown. Statistical significance was assessed by Welch’s *t*-test (*, *P* < 0.05). (**G**) Effect of oxygen concentration on L-to-S conversion. Single colonies of the L1 strain were inoculated into 10 mL of CAMH broth in 50-mL Erlenmeyer flasks containing magnetic stir bars and incubated at 37°C with stirring at 300 rpm for 24 h. Cultures were grown in a multigas incubator set to 0.1% CO_2_ and to the indicated oxygen concentrations (20%, 15%, 10%, or 5%). The proportion of S-type cells was determined by plating and colony counting. Data represent means ± SD from three biological replicates, with individual data points shown. Statistical significance compared to 20% O_2_ was assessed by Welch’s one-way ANOVA followed by Dunnett T3 multiple comparisons test (*, *P* < 0.05; ***, *P* < 0.001).

L-to-S conversion showed some variation across different culture media, while temperature (25°C–42°C) had minimal impact (Fig. S1A and B). In contrast, shaking speed had a strong effect on conversion efficiency: S-type cells consistently emerged at 120 rpm but were nearly absent at 180 rpm (detected in only 1 of 12 cultures at 4.5%, with the remaining 11 cultures showing no detectable S-type cells). Flask culture experiments confirmed this inverse correlation (Fig. 4A). Time-course analysis at 60 rpm showed that S-type cells began to increase notably around 12 h (Fig. 4B), similar to tube culture observations.

Having confirmed that S-type cells emerge and increase under low-shaking conditions, we hypothesized that these cells possess a growth advantage over L-type cells specifically under such conditions. To test this, we first compared the growth of L1 and S1 strains at 180 rpm and 60 rpm (Fig. 4C). At 180 rpm, both strains exhibited similar growth curves and continued proliferating throughout the 9-h incubation. In contrast, at 60 rpm, L1 ceased growth after 6 h, whereas S1 maintained steady proliferation, indicating a divergent response to reduced shaking. To further evaluate this growth advantage, we performed a competition assay using a 1:1 mixture of L1 and S1 strains at both shaking speeds (Fig. 4D). To distinguish the inoculated S1 cells from spontaneously emerging S-type cells, we used S1 carrying the pWH1266 plasmid, which confers resistance to ampicillin and tetracycline (16). At 180 rpm, the growth dynamics of L1 and S1 were largely comparable throughout the 9-h cultivation. In contrast, at 60 rpm, L1 growth plateaued after 6 h, whereas S1 continued to proliferate, consistent with the monoculture growth curves. This resulted in higher relative fitness (*w*) for S1 under low-shaking conditions (*w* = 2.87, SD 0.90 at 6–9 h), supporting that S-type cells gain a growth advantage when oxygen availability is limited.

Because shaking speed influences aeration, we examined whether oxygen limitation promotes L-to-S conversion. Our initial experiments using AnaeroPack showed that low-oxygen conditions substantially increased the emergence of S-type cells (Fig. 4E). Consistent with this, competition assays demonstrated that S-type cells had a clear selective advantage under microaerophilic conditions (Fig. S2). To quantify this effect more precisely, we next assessed the impact of defined oxygen concentrations using a multigas incubator. As cultivation in this incubator requires magnetic stirring rather than orbital shaking, we first tested whether stirring speed affected the conversion frequency. Cultures stirred at 50 rpm produced markedly higher proportions of S-type cells than those stirred at 300 rpm, a pattern similar to that observed under shaking at 60 rpm and 180 rpm, respectively (Fig. 4F). Using a stirring rate of 300 rpm, we then examined the effect of controlled oxygen concentrations. Lowering the oxygen concentration to 10% or 5% greatly increased the proportion of S-type cells (Fig. 4G). These findings demonstrate that L-to-S conversion is strongly promoted under oxygen-limited conditions in a dose-dependent manner.

### L-to-S conversion mutations arise during liquid culture

Because S-type cells became detectable as early as 12 h after inoculation under low-shaking conditions, we initially hypothesized that L-to-S conversion mutations might arise within L-type colonies prior to liquid culture. PCR screening using primers specific for IS*Aha2* insertions in *wzy* (Table 1 and Text S1) detected amplification products in multiple L1 colonies (Fig. S3A and B), and sequencing confirmed IS*Aha2* insertions at various positions and orientations (Fig. S3C), demonstrating the presence of mutation-carrying subpopulations within apparently homogeneous L-type colonies. However, dilution plating of several L-type colonies failed to detect S-type colonies among over 10,000 screened colonies, suggesting that such pre-existing mutants remain at extremely low frequencies.

**Fig 5:**
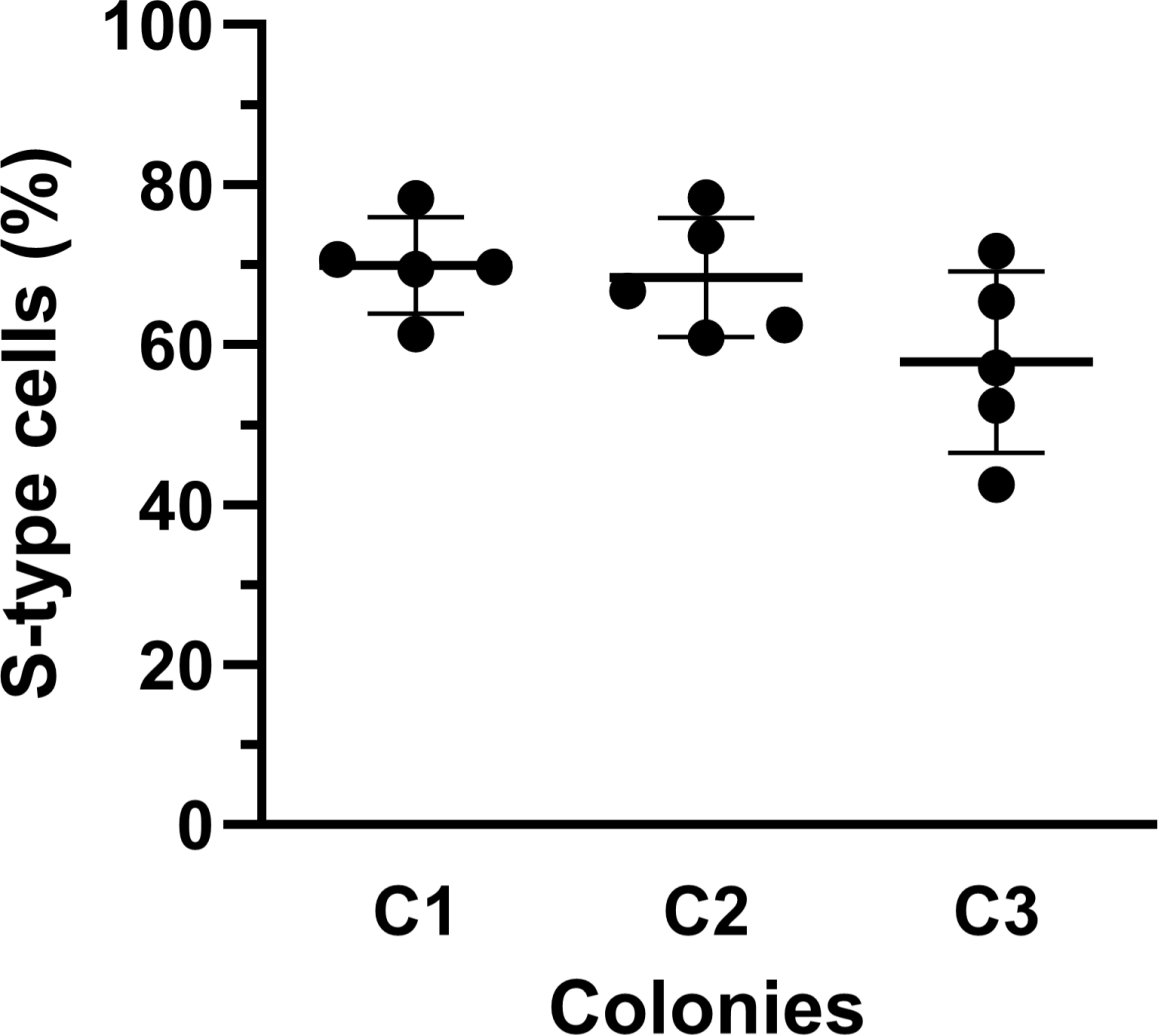
*De novo* L-to-S conversion mutations arise during liquid culture. Cells from three independent L-type colonies (C1–C3) were diluted to approximately 100–200 cells/mL and inoculated into five separate test tubes to achieve 5–10 cells per tube, each containing CAMH broth. After incubation at 37°C with shaking at 120 rpm for 24 h, the proportions of S-type cells in each tube were determined by colony morphology. Each dot represents an independent culture tube, and horizontal lines indicate the mean value for each colony. S-type cells reproducibly emerged in all cultures, reaching 40%–80% of the total population after 24 h.

To determine whether *de novo* mutations also arise during liquid culture, we performed cultivation experiments using highly diluted cell suspensions. Cells from three independent L-type colonies were diluted to approximately 100–200 cells/mL, and 50 μL aliquots (equivalent to 5–10 cells) were inoculated into five separate test tubes containing CAMH broth. Given that S-type variants comprise less than 1 in 10,000 cells within L-type colonies, the probability of S-type cells being present in these inocula was negligible. Consistent with this, direct plating of 500 μL of the diluted suspension (equivalent to 50–100 cells) yielded no S-type colonies. Nevertheless, after 24 h of cultivation at 120 rpm, S-type cells emerged in all five tubes from each of the three colonies, constituting 40%–80% of the population (Fig. 5). These findings demonstrate that L-to-S conversion mutations arise *de novo* during liquid culture at sufficiently high rates to account for the observed phenotypic shift. While pre-existing mutations within colonies may contribute to the rapid emergence of S-type cells, they are not required for the conversion process.

## DISCUSSION

In this study, we characterized a clinical *A. baumannii* isolate that spontaneously gives rise to two morphologically and functionally distinct variants: (i) an L type with a hypercapsule and (ii) an S type with reduced or absent capsule. The conversion from L to S occurred reproducibly under oxygen-limited conditions and was driven by multiple independent mutations within the *cps* gene cluster, including IS insertions and a single-nucleotide deletion. This phenotypic shift was associated with a trade-off between protective traits and environmental adaptability. While phenotypic switching has been previously reported in *A. baumannii* strain AB5075, that system is mediated by transcriptional regulation and is fully reversible, generating opaque and translucent colony types with differing virulence and resistance (17–21). In contrast, the L-to-S conversion observed in OCU_Ac16b is mutation-driven and unidirectional under our experimental conditions, reproducibly yielding S-type variants during liquid culture. These findings uncover a distinct, mutation-based mechanism of capsule variation that expands our understanding of how *A. baumannii* generates phenotypic diversity to adapt to environmental challenges.

Capsule biosynthesis loci in *A. baumannii* are frequently disrupted by ISs, as revealed by genome-wide surveys (22). Additionally, frameshift-like sequence anomalies have been identified in the *cps* locus in many genome assemblies, although their biological validity remains unclear (23). However, the functional and phenotypic consequences of such disruptions have remained largely unexplored. In this study, we provide experimental evidence that IS-mediated and point mutation-driven disruptions within the *cps* cluster reproducibly give rise to capsule-deficient variants. This demonstrates a dynamic mechanism of capsule switching that facilitates the rapid emergence of mutants with distinct survival advantages. Although our study focused on capsule loss, previous work using a different *A. baumannii* strain showed that a capsule-deficient state caused by an IS insertion in *itrA* can revert via scarless excision under specific conditions (22). These findings raise the possibility that *A. baumannii* may utilize both capsule loss and reversion as a reversible switching system to enhance environmental adaptability.

Similar mutation-driven capsule switching has also been observed in *Klebsiella pneumoniae*. Chiarelli et al. demonstrated that carbapenemase-producing *K. pneumoniae* clinical isolates frequently undergo mucoid-to-nonmucoid switching through diverse genetic events, including IS insertions (24). Unlike *A. baumannii* OCU_Ac16b, in which capsule-deficient variants do not visibly expand within colonies on agar plates, the nonmucoid mutants of *K. pneumoniae* rapidly grow within colonies to form distinguishable nonmucoid sectors. Accumulating evidence from both clinical and laboratory settings indicates that *K. pneumoniae* employs capsule switching as an integral strategy to survive in diverse environments (25–29). These parallel findings in *A. baumannii* and *K. pneumoniae* suggest that mutation-driven capsule switching may represent a shared adaptive strategy among encapsulated bacteria, facilitating phenotypic plasticity in response to environmental challenges.

Our results highlight the potential impact of capsule switching on clinical diagnostics and infection outcomes. In routine clinical diagnostics, phenotypic or susceptibility testing is typically performed on a single colony isolated from a patient sample. If phenotypically distinct variants such as L- and S-type cells coexist within the same strain, this practice may lead to incomplete or misleading conclusions regarding the strain’s resistance or virulence profile. Beyond this diagnostic consideration, understanding the distinct characteristics of each variant is also important for predicting their behavior in clinical settings. For example, the desiccation tolerance of L-type cells may support prolonged survival on dry surfaces, facilitating environmental persistence and nosocomial transmission. Their enhanced resistance to serum killing may promote systemic dissemination once in the host. In contrast, S-type variants exhibit enhanced surface attachment and biofilm formation, favoring colonization of medical devices and persistence in chronic infections. Their superior growth under oxygen-limited conditions may confer a fitness advantage in hypoxic tissue environments such as necrotic wounds or inflamed lung tissue. These complementary traits suggest that reversible switching between L and S forms enables *A. baumannii* to exploit diverse niches within different anatomical sites and contributes to its persistence and success in clinical settings.

While our study provides novel insights into the mechanism and biological significance of capsule loss in *A. baumannii* OCU_Ac16b, several important questions remain unresolved. It is unclear whether the frequent mutations observed, particularly IS insertions, are specific to the *cps* cluster or whether similar mutations arise elsewhere in the genome but remain undetected—either because they impair growth and are counterselected or because they confer no fitness advantage and thus fail to expand under the given culture conditions. The extent to which such mutations are reversible, either through IS excision or reversion mutations such as single-nucleotide duplications, also remains to be determined. In addition, the mechanisms underlying the fitness disadvantage of L-type cells under oxygen-limited conditions, as well as the increased β-lactam susceptibility associated with capsule loss, are not yet fully understood. Furthermore, we do not have enough data to assess whether the observed phenomena are unique to OCU_Ac16b or represent a broader strategy among thickly capsulated clinical strains. Finally, we did not directly assess the virulence of the L- and S-type variants in an *in vivo* infection model. Future studies should aim to comprehensively identify S-type mutations, assess their reversibility under relevant selection pressures, and compare gene expression profiles under aerobic and microaerobic conditions, in order to better understand the mechanisms governing capsule switching, including its regulatory control and responsiveness to environmental cues. Evaluating whether other thickly capsulated strains exhibit similar behavior under hypoxia will further clarify the generalizability of our findings. Additionally, *in vivo* virulence assessment using appropriate infection models will be valuable to determine how the observed phenotypic trade-offs translate into distinct infection outcomes and pathogenic potential.

## MATERIALS AND METHODS

### Bacterial strains and growth conditions

The *A. baumannii* strain OCU_Ac16b is a clinical isolate recovered from a patient at Osaka City University Hospital in 2015 (14). *A. baumannii* ATCC 19606^T^, AB5075-UW, and AB10110 served as reference strains. AB10110 is a capsule-deficient derivative of AB5075-UW carrying a T101 transposon insertion in *wzy* (30).

Cultivation was routinely performed in 14-mL round-bottom test tubes or 50-mL Erlenmeyer flasks containing 3 mL or 10 mL of CAMH broth, respectively. For experiments evaluating the proportion of S-type cells during or after 24-h cultivation of L-type cells, the entire single colony was directly inoculated into the medium without prior adjustment of cell density. For experiments requiring OD_600_ or CFU normalization, such as competition assays and biofilm formation and adhesion assays, cells were scraped from agar plates, suspended in 1 mL of CAMH broth, and incubated at 37°C with shaking at 180 rpm for 1 h prior to OD_600_ measurement, unless otherwise specified. We note that an OD_600_ of 1.0 corresponds to approximately 1 × 10^9^ CFU/mL for both L-type and S-type strains under our experimental conditions.

To generate low-oxygen conditions (Fig. 4E; Fig. S2), flasks were placed in sealed plastic pouches together with an AnaeroPack MicroAero (Mitsubishi Gas Chemical Co., Inc., Tokyo, Japan). The pouches were then incubated at 37°C with shaking at 180 rpm. For experiments examining the effect of stirring speed (Fig. 4F), cultures were grown in 50-mL Erlenmeyer flasks containing a magnetic stir bar (20 mm length, 7 mm diameter) with stirring at either 50 rpm or 300 rpm. For experiments examining the effect of oxygen concentration (Fig. 4G), cultures were similarly grown in flasks with magnetic stirring at 300 rpm, but placed in a multigas incubator (SMA-80DRS, Astec Co., Ltd., Fukuoka, Japan) set to 0.1% CO_2_ and the indicated O_2_ concentrations.

### Competition assay

To evaluate the relative fitness of S-type cells compared to L-type cells, L1 and S1 pWH1266 cells were preincubated in CAMH broth for OD_600_ adjustment, as described in the *Bacterial strains and growth conditions* section. Both strains were then inoculated into 10 mL of CAMH broth in 50-mL Erlenmeyer flasks at a 1:1 ratio, with a final concentration of approximately 1 × 10^7^ CFU/mL for each strain. Cultures were incubated at 37°C for 9 h under different conditions: with shaking at either 180 rpm or 60 rpm or at 180 rpm under microaerophilic conditions using AnaeroPack MicroAero. After incubation, bacterial suspensions were appropriately diluted and plated on CAMH agar with or without tetracycline (10 µg/mL) and ampicillin (100 µg/mL) to determine the proportion of S-type colonies.

Relative fitness (*w*) of S-type cells was calculated using the equation, *w* = [*x*_2_(1 − *x*_1_)] / [*x*_1_(1 − *x*_2_)], where *x*_1_ is the initial frequency of S-type cells and *x*_2_ is the final frequency (31).

### Genome sequencing and mutation identification

Cells grown overnight on CAMH agar plates were harvested, and genomic DNA was extracted using Genomic-tip 100/G (Qiagen). Whole-genome sequencing of L1 and S1 was performed using PacBio Sequel IIe and Illumina HiSeq X platforms. Strains S2–S5 were sequenced on the Illumina NovaSeq 6000 platform. L1 was assembled *de novo* with Flye v2.9.2 (32) and polished with Pilon v1.24 (33). S1 was assembled by mapping to L1 using Minimap2 (34). Mutations were identified using breseq (35) for S1 and reference-based variant calling for S2–S5. Detailed methods are provided in the Supplemental Material.

During genome analysis, we identified three plasmids: pOCU_Ac16a_1, pOCU_Ac16a_3, and pOCU_Ac16a_4, the latter having been missed in our previous analysis of OCU_Ac16a.

### Antimicrobial susceptibility tests

Antimicrobial susceptibility testing was performed using Etest strips (bioMérieux, Marcy-l’Étoile, France) in accordance with the guidelines of the Clinical and Laboratory Standards Institute.

### Serum resistance assay

Human blood was collected from three healthy donors (all coauthors of this study), combined, and used to prepare pooled serum. The blood was left at room temperature for 2 h to allow clotting and then centrifuged at 2,330 × g for 5 min. The resulting serum was aliquoted and stored at −80°C until use. Heat-inactivated serum was prepared by incubation at 56°C for 30 min.

For the serum resistance assay, pooled serum was diluted with PBS to final concentrations of 2.5% and 10%. Cells of the tested strains were inoculated from single colonies into CAMH broth, cultured at 180 rpm for 6 h, and then washed twice with PBS. The resulting cell suspension was adjusted to an OD_600_ of 1.0. A 10-μL aliquot of this suspension (~1  ×  10^7^ CFU) was added to 200 μL of diluted serum in a 96-well plate and incubated at 37°C with shaking at 100 rpm for 2 h. After incubation, the mixtures were serially diluted and plated to determine CFU, and survival rates were calculated.

### Biofilm formation and adhesion assays

For biofilm formation assays, cells preincubated in CAMH broth for OD_600_ adjustment, as described in the *Bacterial strains and growth conditions* section, were inoculated into 1 mL of BHI medium in each well of 24-well polystyrene plates at an initial OD_600_ of 0.01. The plates were incubated under static conditions at 37°C for 48 h. After incubation, the culture medium was discarded, and the wells were washed once with PBS. Biofilms were stained with 2 mL of 0.1% crystal violet for 20 min, washed three times with PBS, and air-dried. The dye was then solubilized with 2 mL of 95% ethanol, and the absorbance was measured at 590 nm.

For adhesion assays, cells were inoculated into 1 mL of CAMH broth per well in 24-well plates at an initial OD_600_ of 0.1 and incubated at 37°C with shaking at 100 rpm for 2 h. After incubation, the medium was discarded, and the wells were gently washed twice with PBS to remove planktonic cells. Attached cells were stained and quantified as described for the biofilm assay.

### Desiccation tolerance assay

Cells were pre-cultured in CAMH broth at 37°C with shaking (180 rpm) for 3 h, washed three times with distilled water, and resuspended to an OD_600_ of 0.1. Ten microliters of suspension was dispensed into 96-well plates and incubated at 36°C under 13 ± 3% relative humidity. At designated time points (1, 2, 3, 4, 5, 12, or 21 days), cells were rehydrated with 100 μL distilled water and incubated at 36°C for 30 min. After resuspension by pipetting, samples were serially diluted and plated to determine CFU.

### Density gradient centrifugation assay

Density gradient centrifugation was performed essentially as described previously (36). Cells grown overnight on CAMH agar plates were collected using a 10-µL disposable loop from a confluent lawn and resuspended in 10 mL of CAMH broth. To disperse bacterial aggregates, the suspension was incubated at 37°C with shaking at 180 rpm for 1 h. After washing twice with PBS, the cells were resuspended in 500 µL of PBS. Percoll (P4937; Sigma-Aldrich, St. Louis, MO) was diluted to 40% (vol/vol) with PBS, and 500 µL was transferred to a 2-mL microtube. The bacterial suspension (100 µL) was gently layered on top of the Percoll solution. Density gradient centrifugation was performed at 9,000 × g for 30 min at room temperature, and the distribution of cells was visually inspected.

### Transmission electron microscopy

TEM was performed as described previously (17) with minor modifications. Cells from CAMH agar plates were fixed in 0.1 M sodium cacodylate buffer (pH 7.4) containing 2% paraformaldehyde, 2.5% glutaraldehyde, 0.075% ruthenium red, and 1.55% L-lysine acetate for 20 min on ice, washed twice with 0.1 M sodium cacodylate buffer containing 0.075% ruthenium red, and incubated in the same fixative lacking L-lysine acetate for 2 h on ice. After two washes with sodium cacodylate/ruthenium red buffer, samples were post-fixed in the same buffer containing 1% osmium tetroxide for 1 h at room temperature, washed twice with distilled water, and embedded in 3% agar. The agar blocks were dehydrated in a graded ethanol series, infiltrated with propylene oxide, then with increasing ratios of propylene oxide and epoxy resin (Quetol 812), and polymerized at 60°C for 72 h. Ultrathin sections (~70 nm) were cut with an ultramicrotome (Ultracut UCT; Leica Microsystems), stained with 5% uranyl acetate in 50% ethanol, followed by Reynolds lead citrate, and examined under a TEM (Talos F200C G2; Thermo Fisher Scientific) operated at 200 kV.

### Statistical analysis

Statistical analyses were performed using GraphPad Prism version 10 (GraphPad Software, San Diego, CA). The specific statistical tests applied to each data set are indicated in the corresponding figure legends. Briefly, Welch’s *t*-test was used for pairwise comparisons, and Welch’s one-way ANOVA followed by Dunnett T3 multiple comparisons test was used for multigroup comparisons. For time-course data in Fig. 4D, two-way repeated-measures ANOVA was performed, with the Geisser–Greenhouse correction applied to account for violations of sphericity. All tests were two-tailed, and *p* values of < 0.05 were considered statistically significant.

### Use of generative AI tools

During the manuscript preparation process, generative AI tools (ChatGPT by OpenAI and Claude by Anthropic) were used in a limited capacity to assist with English language editing and phrasing suggestions. All scientific content, data interpretation, and conclusions are solely the work of the authors.

## Data Availability

The complete genome sequences of the *A. baumannii* strains L1 and S1, including the chromosome and three plasmids (pOCU_Ac16a_1, pOCU_Ac16a_3, and pOCU_Ac16a_4), have been deposited in the DDBJ/EMBL/GenBank databases under accession numbers AP043679–AP043686. The DDBJ entry for the related strain OCU_Ac16a (AP023077–AP023080) has been updated to include the additional plasmid pOCU_Ac16a_4 (AP043958). Raw sequencing data have been deposited in the DDBJ Sequence Read Archive under accession numbers DRX698961–DRX698968 (PacBio and Illumina data for L1 and S1, and Illumina data for S2–S5).

## AFTER EPUB

*[This article was published on 29 December 2025 with errors in Acknowledgments. The Acknowledgments were corrected in the current version, posted on 5 January 2026.]*

## SUPPLEMENTAL MATERIAL

Supplemental material mbio.02366-25-s0001.docx Text S1 and Fig. S1 to S3.

ASM does not own the copyrights to Supplemental Material that may be linked to, or accessed through, an article. The authors have granted ASM a non-exclusive, world-wide license to publish the Supplemental Material files. Please contact the corresponding author directly for reuse.
