# Supplementary material for: Benefits and costs of a hypercapsule and the mechanism of its loss in a clinical isolate of Acinetobacter baumannii
Source: mBio. 2025 Dec 29;17(2):e02366-25. doi: 10.1128/mbio.02366-25 (PMC12892936; doi:10.1128/mbio.02366-25)
Supplement: Supplemental material — Text S1 and Fig. S1 to S3. [file mbio.02366-25-s0001.docx]

**Supplemental Material**

**Text S1. Supplemental Methods**

**PCR screening for *wzy*::IS*Aha2* insertions in individual L-type colonies**

To detect IS*Aha2* insertions in *wzy* within single colonies, nested PCR was performed as illustrated in Fig. S3. Colonies were obtained by directly streaking glycerol stocks onto agar plates and incubating overnight (~18 hours) at 37°C. Entire well-isolated colonies were resuspended in 30 µL of sterile water and heat-treated at 99°C for 15 minutes. Each sample was then split into two 15-µL aliquots, both of which were used in separate first-round PCR reactions targeting opposite orientations of IS*Aha2* insertions. First-round PCR was conducted using MightyAmp DNA Polymerase Ver.3 (Takara Bio, Shiga, Japan), followed by nested PCR using Ex Taq polymerase (Takara Bio). Primer sequences are listed in Table 1. PCR products were gel-analyzed, purified, and Sanger sequenced to confirm insertion and determine the site.

**Genome sequencing and bioinformatics analysis**

**Library preparation:**

Genomic DNA extracted from cells scraped from overnight cation-adjusted Mueller Hinton agar plates was used to prepare PacBio and Illumina libraries. For strains L1 and S1, PacBio libraries were constructed using the SMRTbell Express Template Prep Kit 2.0 (Pacific Biosciences, Menlo Park, CA), generating approximately 250 Mb of HiFi reads per sample. Illumina libraries were prepared using the NEBNext Ultra II DNA Library Prep Kit (New England Biolabs, Ipswich, MA) to obtain paired-end 150-bp reads. Strains S2–S5 were sequenced on the Illumina NovaSeq 6000 platform using the NEBNext Ultra II kit.

**Quality control and read processing:**

PacBio HiFi reads were filtered using Filtlong v0.2.1 with a minimum length of 2,000 bp and a minimum quality score of 20, retaining the top 90% of reads (~230 Mb). The N50 values of L1 and S1 were 14,951 bp and 13,931 bp, respectively. Illumina reads were trimmed using fastp v0.23.1 (Supplemental Ref. 1).

**Assembly procedures:**

For L1, filtered HiFi reads were assembled *de novo* into four circular contigs using Flye v2.9.2 (32), and polished twice with Pilon v1.24 (33) using both PacBio and Illumina reads. For S1, HiFi reads were mapped to the L1 genome using Minimap2 (34), and the consensus sequence was polished with Pilon.

**Variant identification:**

Mutations responsible for phenotypic conversion were identified by comparing S-type genomes with L1. For S1, structural and point mutations were detected by using breseq (35) to align the assembled genome with L1. For S2–S5, quality-filtered Illumina reads were used for both *de novo* assembly and reference-based variant analysis. Contigs spanning the *cps* cluster were compared with the L1 reference, and genome-wide variants were identified by read mapping, variant calling, and manual curation.

**Supplemental Figures**


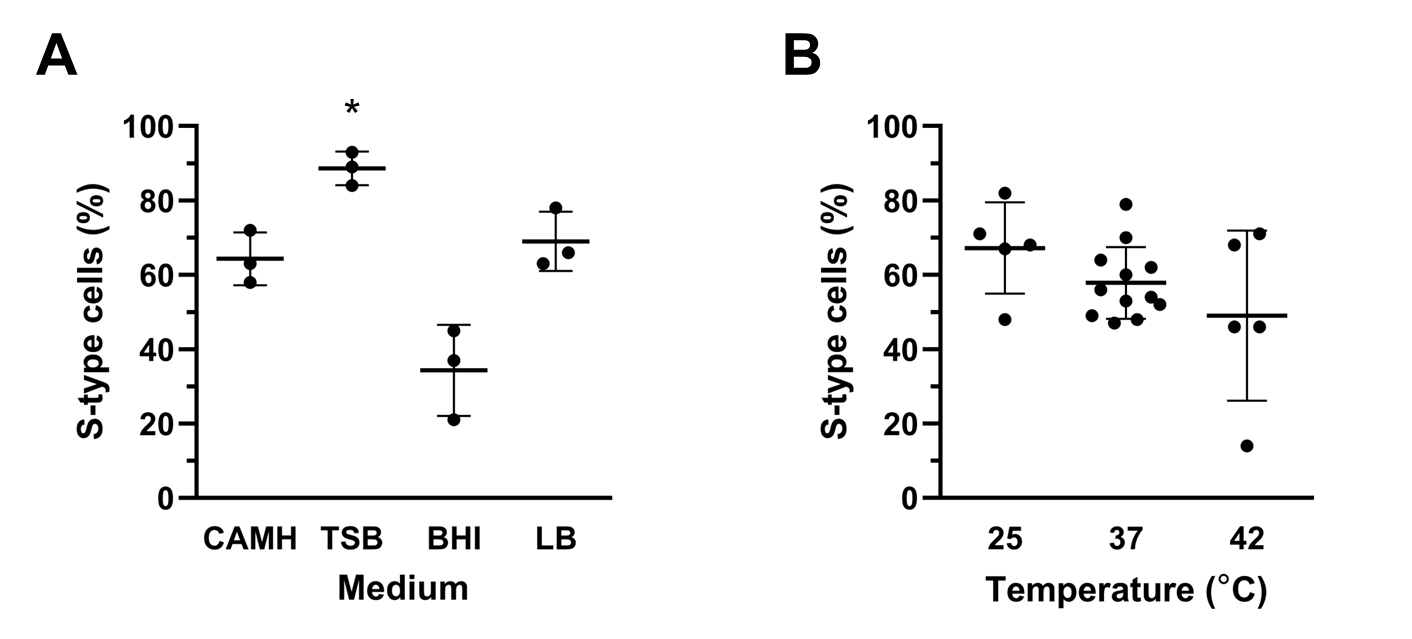


**Fig. S1.** Effects of culture medium and temperature on L-to-S conversion. (A) Effect of culture medium on L-to-S conversion. Single colonies of the L1 strain were inoculated into 3 mL of different media in 14-mL test tubes and incubated at 37°C with shaking at 120 rpm for 24 hours. The proportion of S-type cells was determined by plating and colony counting. Data represent means ± SD from three biological replicates (independent cultures initiated from single colonies), with individual data points shown. Statistical significance was assessed by Welch’s one-way ANOVA followed by Dunnett T3 multiple comparisons test, using CAMH as the control. An asterisk indicates a significant difference compared with CAMH (*, *p* < 0.05). CAMH, cation-adjusted Mueller Hinton broth; TSB, Tryptic Soy Broth; BHI, Brain Heart Infusion broth; LB, Luria-Bertani broth. (B) Effect of temperature on L-to-S conversion. Single colonies of the L1 strain were inoculated into 3 mL of CAMH broth and incubated with shaking at 120 rpm at the indicated temperatures. Cultures were incubated for 24 hours at 37°C and 42°C, and for 48 hours at 25°C due to slower growth at the lower temperature. Data represent means ± SD from five biological replicates at 25°C and 42°C, and twelve biological replicates at 37°C (pooled data from the four independent experiments shown in Fig. 1D), with individual data points shown. Statistical analysis was performed using Welch’s one-way ANOVA followed by Dunnett T3 multiple comparisons test. No significant differences were observed among the temperature conditions.


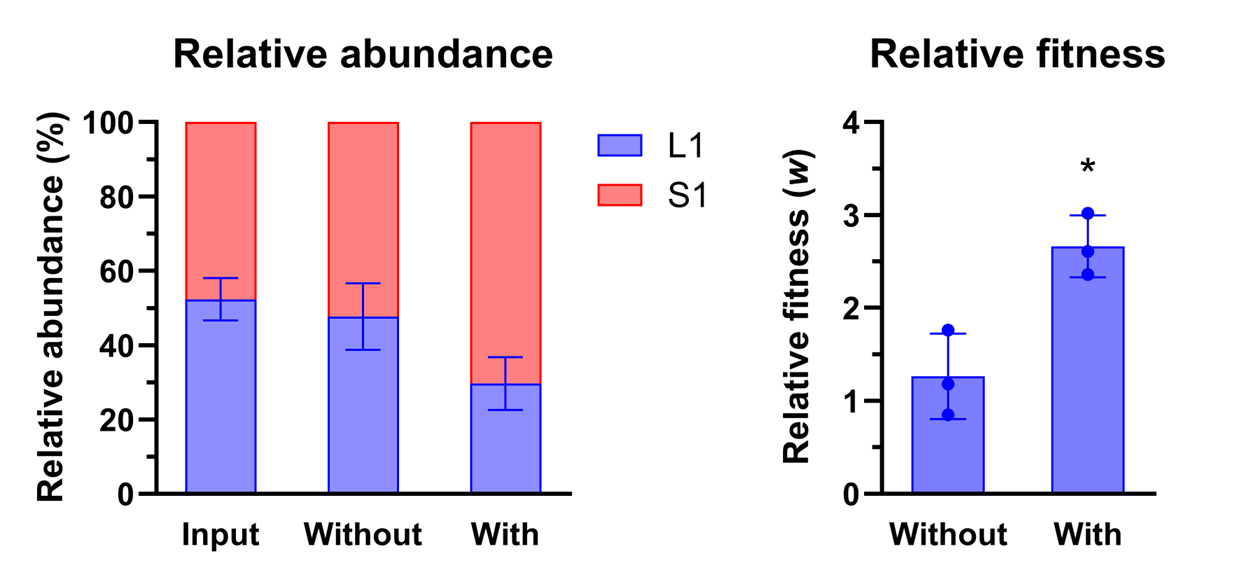


**Fig. S2.** Competition assay under ambient and microaerophilic conditions. L1 and S1 pWH1266 cells were mixed at a 1:1 ratio and cultivated in cation-adjusted Mueller Hinton broth at 37°C with shaking at 180 rpm for 9 hours. Two conditions were tested: "Without" refers to cultivation under ambient conditions, and "With" refers to cultivation in sealed pouches containing AnaeroPack MicroAero to generate microaerophilic conditions. The left panel shows the relative abundance of each strain at input and after 9 hours of cultivation. The right panel shows the relative fitness of S1 cells with respect to L1 cells under the two conditions. Data represent means ± SD from three biological replicates. Statistical significance in the relative fitness data between the two conditions was assessed by Welch’s *t* test (*, *p* < 0.05).


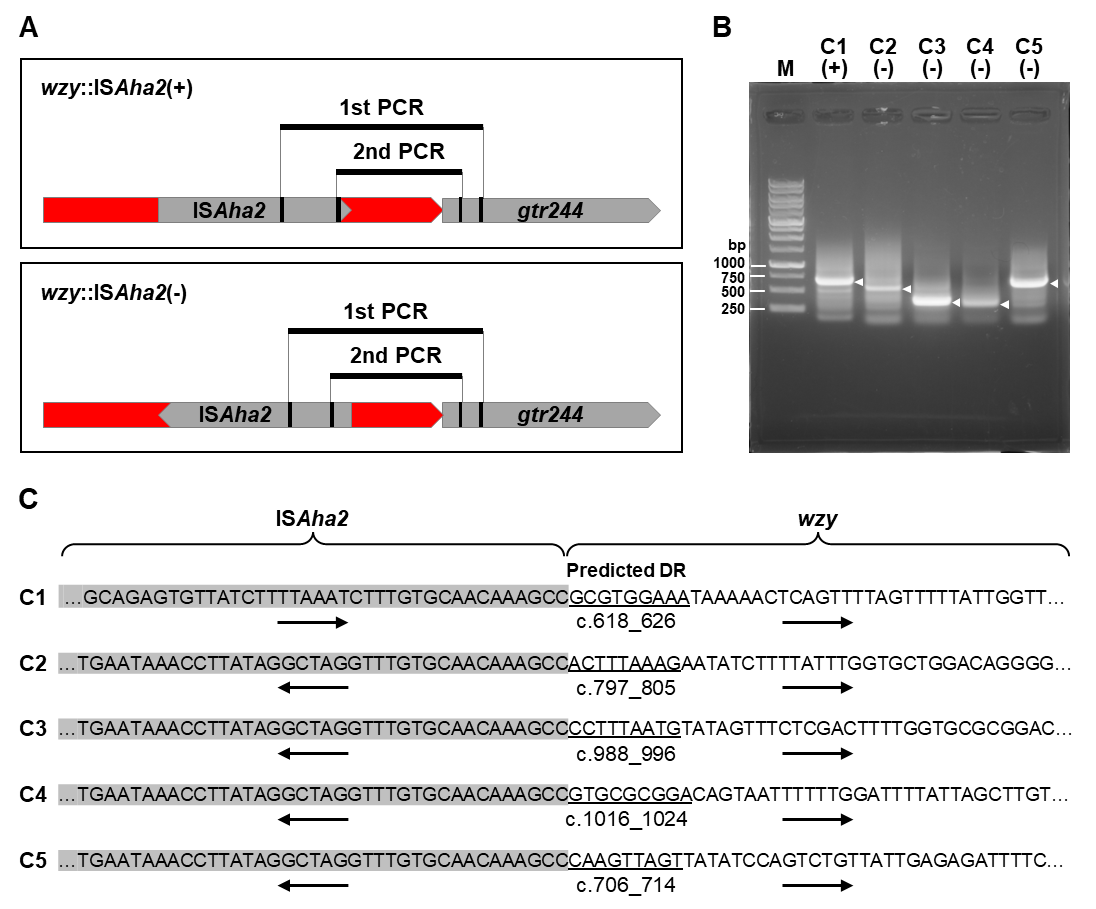


**Fig. S3.** Detection of *wzy*::IS*Aha2* insertions within individual L-type colonies prior to liquid culture. (A) Schematic representation of the nested PCR strategy used to detect IS*Aha2* insertions in *wzy* in both forward (+) and reverse (−) orientations. PCR primers were designed to target sequences within IS*Aha2* and downstream of *wzy* (indicated by black segments), with a first-round PCR followed by a nested second-round PCR to increase specificity. Red arrows indicate *wzy*, and gray elements represent IS*Aha2* and the adjacent gene *gtr244*. (B) Agarose gel electrophoresis showing positive PCR products from nested PCR reactions. Eight individual L-type colonies were tested for IS*Aha2* insertions in both orientations (16 reactions in total), and only the five reactions yielding positive bands (from colonies C1 to C5) are shown. M indicates the molecular weight marker. (+) and (−) denote reactions designed to detect forward or reverse orientations of IS*Aha2* insertions, respectively. White arrowheads indicate bands that were excised and subjected to Sanger sequencing. (C) DNA sequence analysis of the PCR products shown in panel B, illustrating the junctions between IS*Aha2* and *wzy*. Gray highlighting indicates the terminal sequences of IS*Aha2*. Predicted direct repeat (DR) sequences are underlined, and the positions (e.g., c.618_626) refer to nucleotide coordinates within the *wzy* coding sequence corresponding to the DRs. Arrows below the sequences indicate the orientations of IS*Aha2* and *wzy*.

**Supplemental Reference**

1. Chen S, Zhou Y, Chen Y, Gu J. 2018. fastp: an ultra-fast all-in-one FASTQ preprocessor. Bioinformatics 34:i884–i890.
